# Effects of green coffee extract on fasting blood glucose, insulin concentration and homeostatic model assessment of insulin resistance (HOMA-IR): a systematic review and meta-analysis of interventional studies

**DOI:** 10.1186/s13098-019-0489-8

**Published:** 2019-11-05

**Authors:** Omid Nikpayam, Marziyeh Najafi, Samad Ghaffari, Mohammad Asghari Jafarabadi, Golbon Sohrab, Neda Roshanravan

**Affiliations:** 1 Student Research Committee, Tabriz University of Medical Sciences, Tabriz, Iran, Tabriz, Iran; 2 Clinical Nutrition and Dietetics Department, Faculty of Nutrition Sciences and Food Technology, Shahid Beheshti University of Medical Sciences, Tehran, Iran; 3 Cardiovascular Research Center, Tabriz University of Medical Sciences, Tabriz, 5166615573 Iran; 4 Department of Statistics and Epidemiology, Faculty of Health, Tabriz University of Medical Sciences, Tabriz, Iran; 5 Road Traffic Injury Research Center, Tabriz University of Medical Sciences, Tabriz, Iran; 6 Clinical Nutrition and Dietetics Department, Faculty of Nutrition Sciences and Food Technology, National Nutrition and Food Technology Research Institute, Shahid Beheshti University of Medical Sciences, 46, Hafezi St, Farahzadi Blvd, Shahrak Qods, 193954741, Tehran, Islamic Republic of Iran

**Keywords:** GCE, FBG, Insulin, HOMA-IR, Meta-analysis

## Abstract

Many studies have investigated the relationship between coffee and diabetes. Evaluation of the current evidence on the effect of coffee intake on diabetes is critical. Therefore, we aimed to investigate the potential association between green coffee extract (GCE) and fasting blood glucose (FBG), insulin and homeostatic model assessment of insulin resistance (HOMA-IR) by pooling together the results from clinical trials. PubMed, Scopus and Google Scholar were searched for experimental studies which have been published up to December 2018. Randomized controlled trials (RCTs) that investigated the effect of GCE supplementation on FBG, insulin and HOMA-IR in adults were included for final analysis. A total of six articles were included in the meta-analysis. Results revealed that GCE supplementation reduced FBG level (SMD: −0.32, 95% CI − 0.59 to − 0.05, P = 0.02) but had no effect on insulin levels (SMD: −0.22, 95% CI −0.53 to 0.09, P = 0.159). Although analysis showed that GCE supplementation cannot change the HOMA-IR status (SMD: −0.30, 95% CI −0.73 to 0.13, P = 0.172), after stratified studies by GCE dosage (< 400 mg/day versus > 400 mg/day) there was a significant decrease in HOMA-IR status in a dose greater than 400 mg. These findings suggest that GCE intake might be associated with FBG improvement.

## Background

Diabetes mellitus has now reached an epidemic level in both developing and developed countries. Due to the pandemic level and macro- and micro-vascular complications caused by diabetes mellitus, it could emerge as a worldwide issue with huge economic and social costs [1]. Dietary approaches can be a successful strategy to diminish the risk of diabetic complications [2]. Coffee, a main source of nutraceuticals with antioxidant properties, is a commonly consumed beverage around the world with an approximate production of around 60 kg bags in 2017 [3, 4]. Coffee contains at least 1000 compounds with the majority of them found in phenolic components such as chlorogenic acid (CGA) [5]. CGAs belong to a family of esters such as quinic acid and several hydroxyl cinnamic acids (caffeic, ferulic and coumaric acids). The antioxidant potential of coffee can be mainly attributed to its CGAs components [6, 7]. The processing of coffee into roasted coffee generally destroys considerable amounts of CGAs, and due to this fact green coffee, in the form of unroasted beans, has been more widely considered [8]. The anti-inflammatory, anti-oxidant and anti-cancerous effects of green coffee are attributed to CGAs [9, 10]. Most observational research on coffee consumption showed improved glucose homeostasis and lipid profiles [6, 11, 12]. Chlorogenic acid seems to have hypoglycemic effects similar to metformin by improving insulin resistance, evidently without adverse effects [13]. However, clinical trials on the glucose lowering effects of GCE and CGA are controversial and contradictory. Several studies have indicated that GCE supplementation is inversely related to hyperglycemia and insulin resistance [5, 10, 14, 15], In contrast, some studies did not support these outcomes [16, 17]. The objective of this current systematic review and meta-analysis is to investigate the results of human clinical trials assessing the efficacy of GCE as a glucose lowering agent.

## Methods

### Search strategy

This meta-analysis has been carried out according to the preferred reporting items for systematic reviews and meta-Analysis (PRISMA) statement (Picot et al., 2012). All studies were detected by searching in Scopus (http://www.scopus.com), Pubmed (http://www.pubmed.com), ISI web of science (http://www.webofscience.com) up to December 2018. The purpose of the search strategy in this study was to investigate the effect of GCE on glycemic control and the search keywords included: coffee or green coffee or green coffee extract or chlorogenic acid and fasting plasma glucose or fasting blood glucose or fasting blood sugar or glucose or glucose intolerance postprandial or plasma glucose or blood glucose. No restrictions were applied for identifying human studies or clinical trials and relevant studies. Thereupon all the selected studies were chosen with our own inclusion criteria

### Study selection

All single- or double-blind RCT in human samples which investigate the effect of GCE on fasting blood glucose (FBG), insulin and HOMA-IR (homeostatic model assessment of insulin resistance) were included. All of the studies were screened by two independent investigators.

The inclusion criteria included the following:

- 1. Single- or double-blind RCT.
- 2. Human participants over 18 years old.
- 3. Evaluating the effect of GCE on insulin resistance factors (we also included some studies that supplemented another substance with GCE) presented data on FBG, Insulin and HOMA-IR in intervention and placebo groups.

The exclusion criteria were:

- 1. Non RCT trial.
- 2. Trial without control group.
- 3. Duplicate studies and animal studies.
- 4. GCE intake for < 1 month.

### Data extraction

Full texts of all the eligible articles were reviewed following the preferred reporting items for systematic reviews and meta-analyses guidelines [18]. Information included; first author’s name, year of publication, study design, the country of study, number and characteristics of participants in each group, intervention type and dose, duration of intervention, disease type and the GCE impacts on FBG, insulin and HOMA-IR.

### Statistical analysis

Statistical analysis in presented study is performed by STATA software version 12. The effect size of GCE supplement on FBS, insulin and HOMA-IR were investigated through standard mean difference (SMD) with 95% confidence interval (CI) using the inverse variance method, Cohen statistic, and a Random-effects model. Heterogeneity detected by I square (I2) test, significant heterogeneity was defined as I2 > 50% with a P < 0.05. Subgroup analysis was performed to discover source of heterogeneity. To investigate the influence of each study on pooled effect size we used sensitivity analysis. Publication bias among the studies was assessed by funnel plots and also Egger’s regression test and Begg’s test. We converted all standard error (SE) to standard deviation (SD) and SD of mean differences were computed by: SQRT ((SD before2) + (SD after2) − (SD before × SD after)).

## Results

### Included studies

**Fig. 1:**
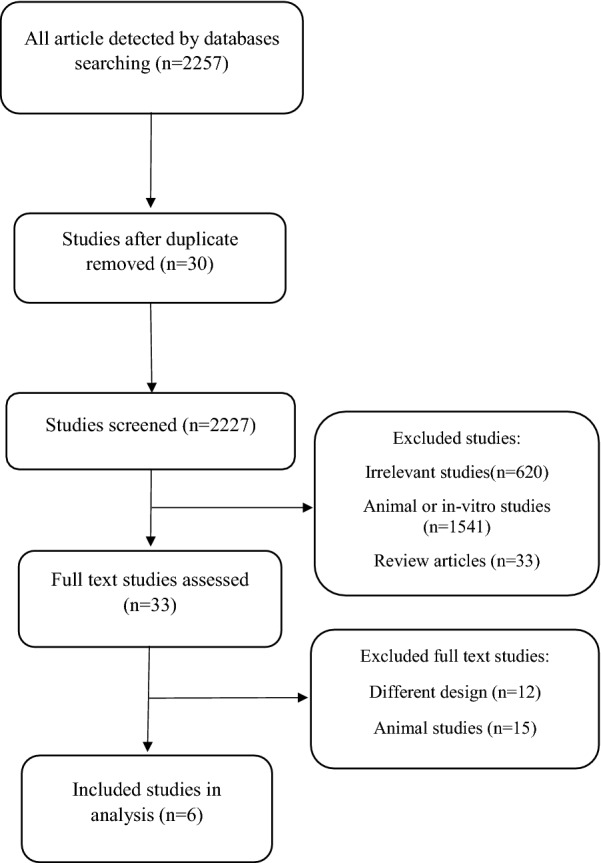
Flow chart of selection study

Firstly, the 2257 articles were detected by searching in Scopus, Pubmed and Web of science. 2194 studies were excluded because of being a duplication, an irrelevant study, animal or in vitro study and review study and then 33 full text papers were revised of which 27 studies, due to different designs and animal samples, were excluded. Eventually, 6 RCT [5, 10, 14–17] met our inclusion criteria and were included in this meta-analysis. The processes of selection and identification papers are presented in Fig. 1.

### Study characteristics

Characteristics of studies are showed in Table 1. Total participant number in all studies was 291, which was included in the analysis. Trials had been published from 2013 to 2018. Four trials were parallel [5, 10, 14, 16] and two studies were cross-over [15, 17]. The duration of intervention among studies varied from 4 to 12 weeks. Six studies reported FBG, 4 insulin [5, 14, 16, 17] and 4 HOMA-IR [5, 14, 16, 17]. The dose of GCE used in different studies was between 100 and 1200 mg. In one study, participants received GCE in supplement to several other extracts. Equality assessment of all trials is presented in Table 2.

**Table 1:** Characteristics of the included study

Author	Country	Year	Participant (n) In/P	Mean age (year) In/P	Total dose (mg)	Study duration (week)	Study population	Study design	Outcome
Zuniga [10]	Mexico	2018	15/15	43/45	1200	12	IGT	RCT	FBS
Roshan 5]	Iran	2018	21/22	52.76/51.95	800	8	MS	RCT	FBS, insulin, HOMA-IR
Shahmohammadi [14]	Iran	2017	22/22	41.36/44.50	1000	8	NAFLD	RCT	FBS, insulin, HOMA-IR
Soga [15]	Japan	2013	18/18	36.1/36.1	329	4	Healthy	Crossover	FBS
Wong [17]	Australia	2014	37/37	58.5/58.5	100	12	BP	Crossover	FBS, insulin, HOMA-IR
Haidari [16]	Iran	2017	30/34	36.1/35.7	400	8	Obesity	RCT	FBS, insulin, HOMA-IR

*In* intervention group, *P* Placebo group, *RCT* randomize clinical trial, *IGT* impaired glucose tolerance, *MS* metabolic syndrome, *NAFLD* non-alcoholic fatty liver disease, *BP* blood pressure, *FBS* fasting blood glucose, *HOMA-IR* homeostatic model assessment of insulin resistance

**Table 2:** Quality assessment of studies

Author (location, year)	Sequence generation	Allocation concealment	Blinding of participants and personnel	Blinding of outcome assessment	Incomplete outcome data	Selective outcome reporting	Other potential threats to validity
Zuniga (Mexico, 2018)	L	U	H	U	L	L	U
Roshan (Iran, 2018)	L	L	L	U	L	L	U
Shahmohammadi (Iran, 2017)	L	U	H	U	L	H	U
Soga (Japan, 2013)	U	U	U	U	L	H	U
Wong (Australia, 2014)	U	U	U	U	L	H	U
Haidari (Iran, 2017)	L	U	U	U	L	H	U

*L* low risk, *U* unclear risk, *H* high risk

### Effects of GCE on FBG

**Fig. 2:**
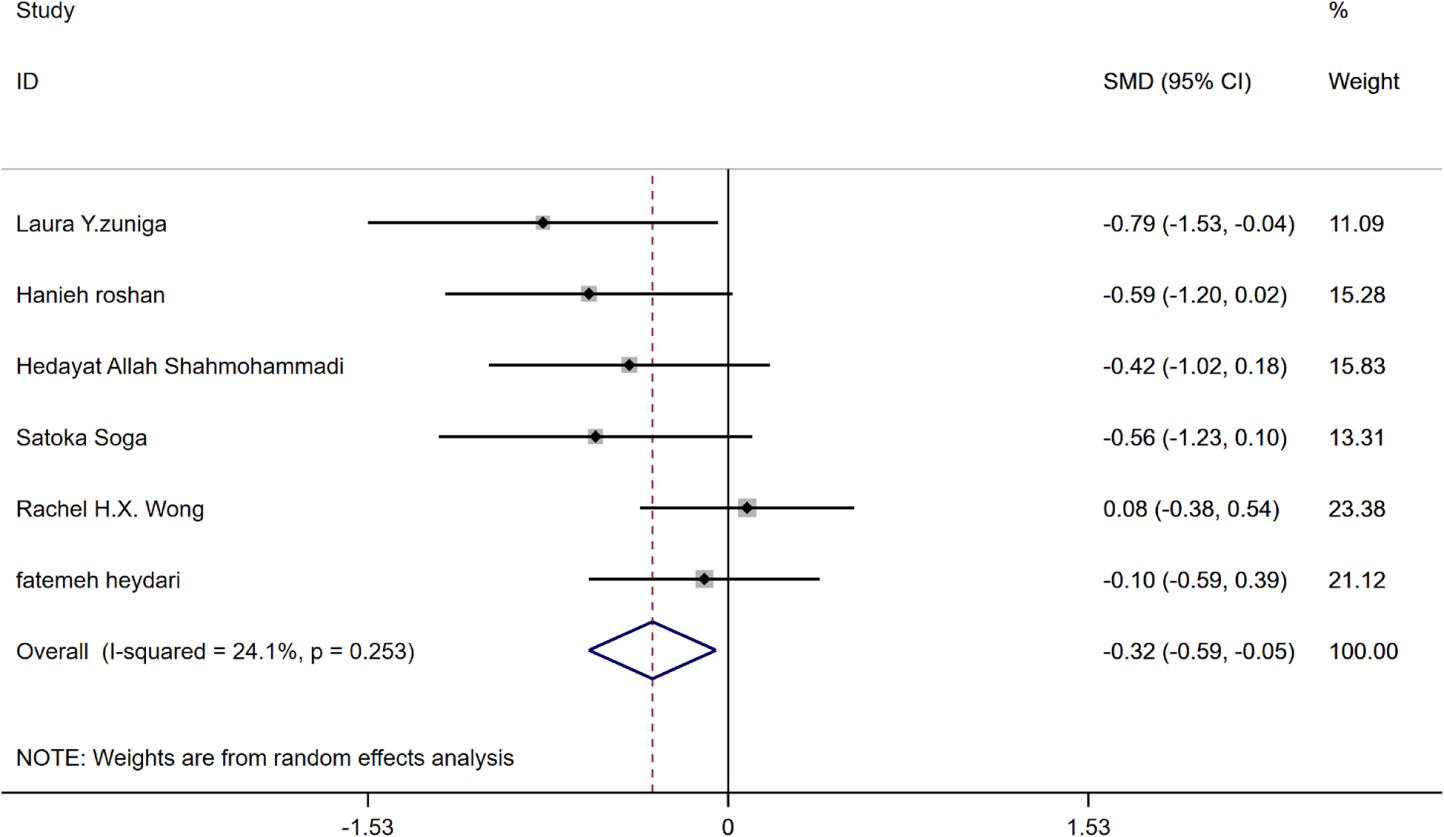
Forest plot of the effect of GCE Supplementation on FBS

**Fig. 3:**
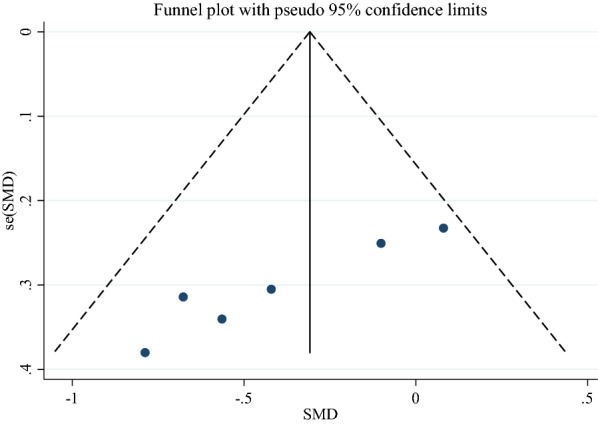
Funnel plot of studies evaluate FBS

Effect of GCE supplementation on FBG is showed in Fig. 2. Findings showed that GCE significantly reduced FBG (SMD: − 0.32, 95% CI − 0.59 to − 0.05, P = 0.02), there was no heterogeneity between studies (I2: 24.1%, P = 0.253). Funnel plot of studies are presented in Fig. 3. According to the figures there was no publication bias between studies (Begg’s P = 0.19 and Egger’s P = 0.11). Sensitivity analysis was conducted to consider the effect of each study on overall SMD. This test revealed that by excluding any one study there was no significant effect.

### Effect of GCE on insulin

**Fig. 4:**
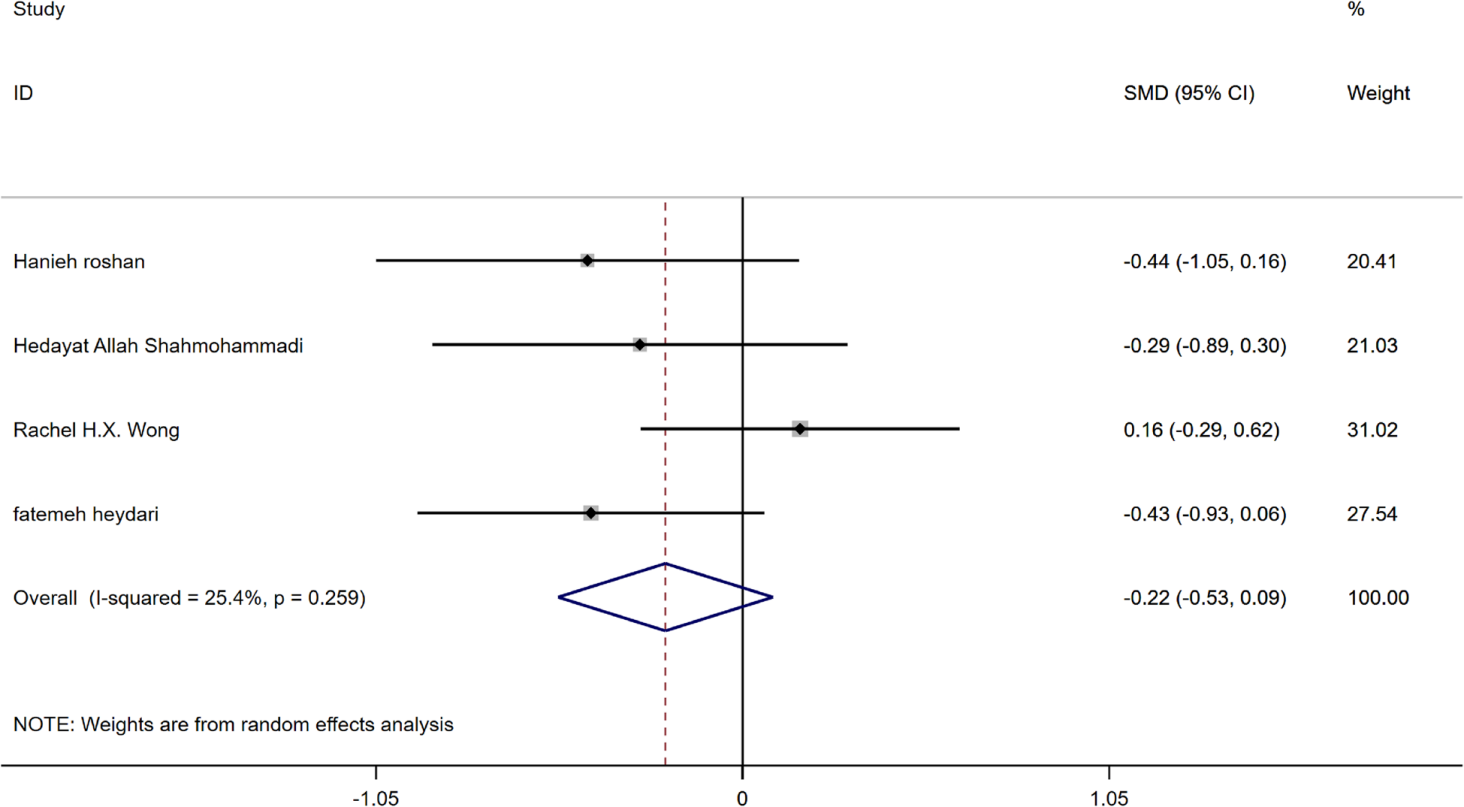
Forest plot of the effect of GCE supplementation of insulin

**Fig. 5:**
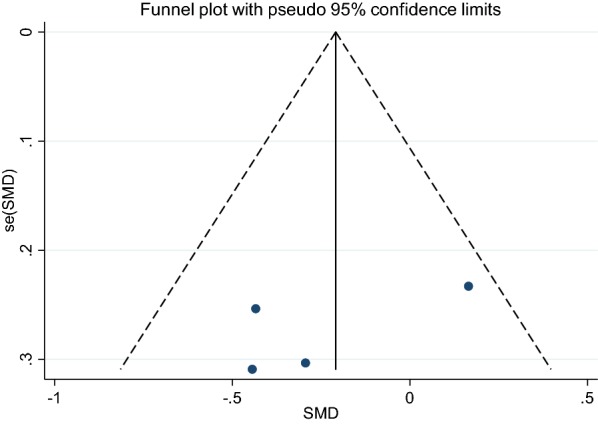
Funnel plot of studies considering insulin

Findings indicated that supplementation with GCE had no effect on insulin (SMD:−0.22, 95% CI −0.53 to 0.09, P = 0.159), this is shown in Fig. 4. According to the I square test (I2:25.4%, P = 0.259) no heterogeneity was observed between studies. The funnel plot of insulin is presented in Fig. 5. According to the shape of the funnel plot, the studies did not have publication bias (Begg’s P = 0.21 and Egger’s P = 0.16). Sensitivity analysis results indicated that by excluding any of the studies SMD was not significantly changed.

### Effect of GCE on HOMA-IR

**Fig. 6:**
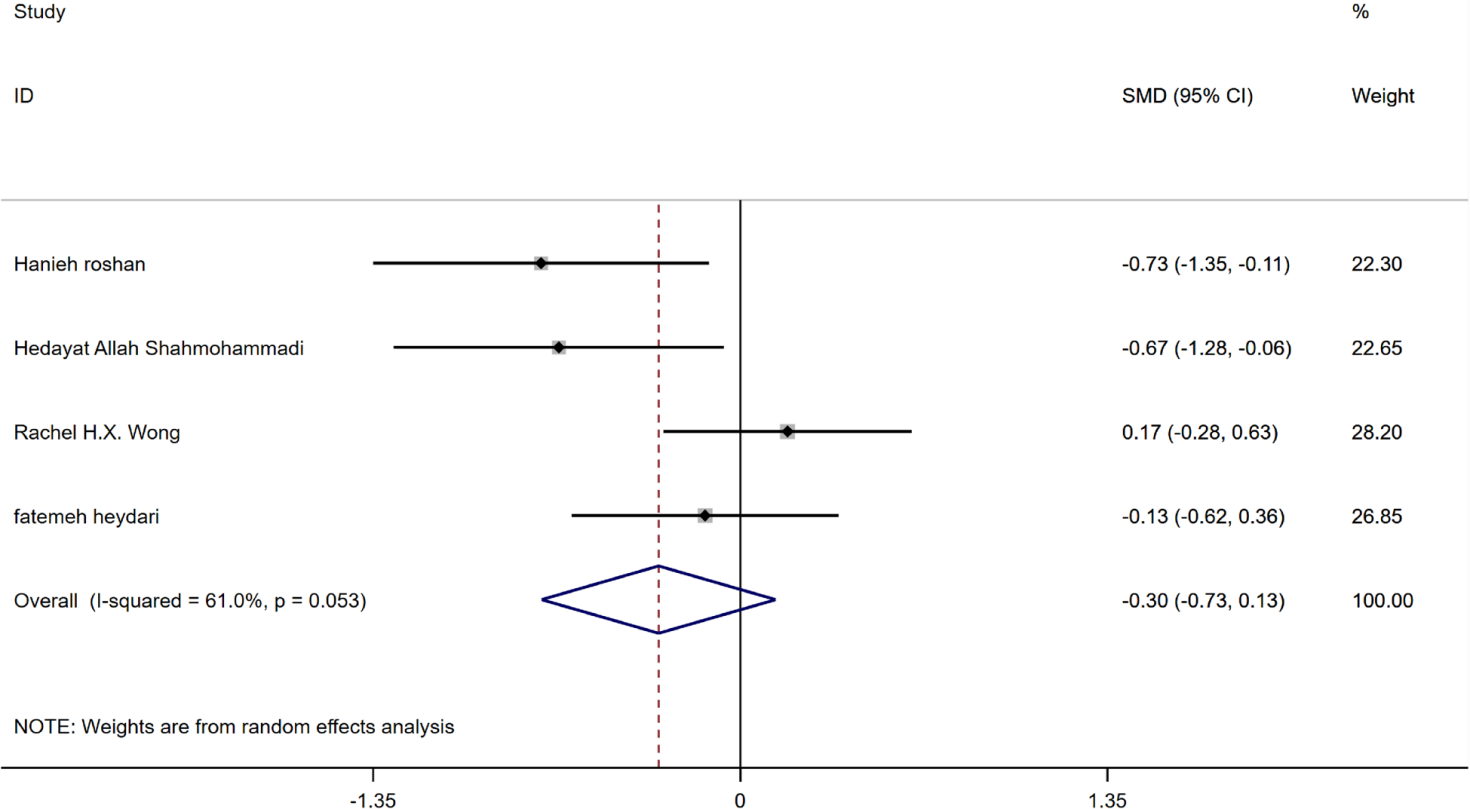
Forest plot of the effect of GCE supplementation of HOMA-IR

**Fig. 7:**
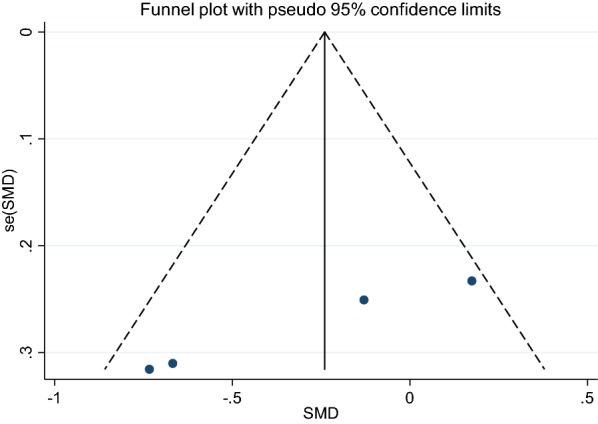
Funnel plot for studies assessment HOMA-IR

Analysis of data showed that supplementation with GCE has no significant effect on HOMA-IR status. (SMD: −0.30, 95% CI −0.73 to 0.13, P = 0.172) Furthermore, there was heterogeneity between studies when considering the effect of GCE on HOMA-IR status (I2:61%, P = 0.053), as shown in Fig. 6. Therefore studies were classified according to the dose used and then analyzed. Subgroup analysis showed that HOMA-IR status in a dose greater than 400 mg of GCE significantly decreases (SMD:−0.7, −1.13 to −0.27, P = 0.002, I2: 0.0%) while its desirable effect was not found in studies that used less than 400 mg GCE, (SMD: 0.03, 95% CI −0.3 to 0.37, I2:0.0%), it has been presented in Table 3. As shown in Fig. 7, funnel plot results did not show any publication bias between studies (Begg’s P = 0.17 and Egger’s P = 0.11). Sensitivity analysis for studies which take the HOMA-IR into consideration are shown by omitting each study which had no change in the overall results.

**Table 3:** Results of subgroup ana lysis of the included randomized controlled trials in meta-analysis of GCE on HOMA-IR

Variables		Dose	
		< 400 mg	> 400 mg
HOMA-IR			
No. of comparison		2	2
SMD		0.03	− 0.7
95% CI			
Lower		− 0.3	− 1.13
Higher		0.37	− 0.27
P value		0.844	0.002
I^2^ (%)		0.0%	0.0%
p-heterogeneity		0.375	0.882

## Discussion

The main purpose of this systematic review was to evaluate the efficacy of GCE as a glucose and insulin resistant lowering supplement. This overall meta-analysis revealed a significant reduction in FBG in GCE compared to the placebo group. However, supplementation with GCE had no effect on insulin and HOMA-IR. Also, there was heterogeneity between studies when considering the effect of GCE on HOMA-IR status. To our knowledge, this is the first meta-analysis that examined the effect of GCE supplementation on the FBG, insulin and HOMA-IR (Table 4).

**Table 4:** Search strategy

Database	Search strategy	Results
Scopus	(TITLE-ABS-KEY (coffee) OR TITLE-ABS-KEY (green AND coffee) OR TITLE-ABS-KEY (green AND coffee AND extract) OR TITLE-ABS-KEY (chlorogenic AND acid))) AND ((TITLE-ABS-KEY (fasting AND plasma AND glucose) OR TITLE-ABS-KEY (fasting AND blood AND glucose) OR TITLE-ABS-KEY (fasting AND blood AND sugar) OR TITLE-ABS-KEY (glucose) OR TITLE-ABS-KEY (glucose AND intolerance AND postprandial) OR TITLE-ABS-KEY (plasma AND glucose) OR TITLE-ABS-KEY (blood AND glucose)	1038
Pubmed	(“Coffee”[Mesh]) OR coffee[Title/Abstract]) OR green coffee[Title/Abstract]) OR green coffee extract[Title/Abstract]) OR “Chlorogenic Acid”[Mesh]) OR Chlorogenic Acid[Title/Abstract])) AND (((((((((“Glucose”[Mesh]) OR Glucose[Title/Abstract]) OR fasting plasma glucose[Title/Abstract]) OR fasting blood glucose[Title/Abstract]) OR fasting blood sugar[Title/Abstract]) OR glucose intolerance postprandial[Title/Abstract]) OR plasma glucose[Title/Abstract]) OR “Blood Glucose”[Mesh]) OR Blood Glucose[Title/Abstract])	462
Web of science	TOPIC: (coffee) *OR* TOPIC: (green coffee) *OR* TOPIC: (green coffee extract) *OR* TOPIC: (chlorogenic acid) *AND* TOPIC: (fasting plasma glucose) OR TOPIC: (fasting blood glucose) OR TOPIC: (fasting blood sugar) OR TOPIC: (glucose) OR TOPIC: (glucose intolerance postprandial) OR TOPIC: (plasma glucose) OR TOPIC: (blood glucose)	757

There are a few studies which have investigated the FBG lowering effects of GCE. Although most of these trials support the protective activity of GCE against hyperglycemia [5, 10, 14], somewhat controversial results were reported. In a crossover trial by Wong et al. fasting glucose levels and insulin sensitivity (HOMA-Index) were unaffected by 100 mg GCE supplementation in hypertensive patients [17]. In a trial by Haidari et al. 64 obese women aged 20–45 years were randomized to either an intervention group (receiving 400 mg green coffee bean extract) or placebo group (receiving placebo). The 8 weeks intervention had no significant effect on FBG and serum insulin level [16]. The inconsistent findings can be due to differences in diverse factors including study design, population, and doses. Furthermore, chlorogenic acid enriched coffee can influence post-prandial glucose concentration [19].

These beneficial effects of GCE may be attributed to the biologically active components in coffee, such as chlorogenic acid, trigonelline, caffeine and magnesium [20, 21]. Diminishing the intestinal glucose absorption; a mechanism achieved by promoting dispersal of the Na+ electrochemical gradient may be considered the potential of GCE in decreasing blood glucose. This diffusion promotes an influx of glucose into the enterocytes [22]. GCE also inhibits the enzymatic activity of hepatic glucose-6-phosphatase, which is responsible for the formation of endogenous glucose originating from gluconeogenesis and glycogenolysis [23]. Another mechanism which is thought to correspond with the FBG lowering effect of CGA is the stimulation of AMP-activated protein kinase (AMPK) activity. Activation of AMPK leads to increasing GLUT4 translocation to plasma membrane which enhances glucose transport to cells. These pathways lead to peripheral glucose disposal [5].

It has been proposed by Song et al. that insulin resistance may be ameliorated by GCE via decreasing phosphorylation of c-Jun N-terminal kinase which causes the activation of insulin receptor substrate-1 and results in the GLUT4 translocation to adipocyte membrane and as a main result, increases the insulin sensitivity [24]. However, our results showed that supplementation with GCE has no significant effect on insulin and HOMA-IR status.

There were some limitations in our meta-analysis. Most of included studies were performed within ≤ 12 weeks and had a small population size. Variation in consumed dose of GCE (100 and 1200 mg/day) in different studies was another limitation for the study. Furthermore, our included studies evaluated the effect of GCE in various populations; therefore we cannot generalize our findings solely to patients with diabetes. And, as a matter of fact, regarding the limited number of studies (only one study), we couldn’t assess the effect of caffeinated green coffee on glycemic indices.

## Conclusion

Overall, our results suggest that GCE supplementation may improve FBG. However, several important points remain which should be considered. The effect size is small and the clinical relevance of this effect is unknown. More precise trials with longer duration of intervention are essential to determine the efficacy and safety of GCE as a glucose lowering supplement and the mechanism of action in population and disease sub-groups should be further explored. All indications show that conducting a study to investigate the effect of GCE on diabetic patients seems essential.

## Data Availability

Please contact authors for data request.

## Abbreviations

GCE: green coffee extract
FBG: fasting blood glucose
HOMA-IR: insulin and homeostatic model assessment of insulin resistance
CGA: chlorogenic acid
PRISMA: preferred reporting items for systematic reviews and meta-analysis
RCT: randomized controlled trials
